# *De novo* acute myeloid leukemia subtype-M4 with initial trisomy 8 and later acquired t(3;12)(q26;p12) leading to ETV6/MDS1/EVI1 fusion transcript expression: A case report

**DOI:** 10.3892/ol.2014.1784

**Published:** 2014-01-08

**Authors:** WALID AL ACHKAR, ABDULMUNIM ALJAPAWE, THOMAS LIEHR, ABDULSAMAD WAFA

**Affiliations:** 1 Department of Molecular Biology and Biotechnology, Division of Human Genetics, Atomic Energy Commission of Syria, Damascus 6091, Syria; 2 Laboratory of Flow-Cytometry, Department of Molecular Biology and Biotechnology, Division of Mammalians Biology, Atomic Energy Commission of Syria, Damascus 6091, Syria; 3 Jena University Hospital, Institute of Human Genetics, Jena 07740, Germany

**Keywords:** t(3;12)(q26;p13), ETV6/MDS1/EVI1, acute myeloid leukemia M4, trisomy 8

## Abstract

The t(3;12)(q26;p13) translocation is a recurrent chromosomal aberration observed in myeloid malignancies. The translocation results in the generation of the ETV6/myelodysplastic syndrome 1 (MDS1)/ectopic viral integration site 1 (EVI1) fusion gene. However, the present case report is the first to present this rearrangement in acute myelogeneous leukemia (AML)-M4. Notably, this case is the first report of AML-M4 with an initial trisomy 8 and secondary acquired t(3;12)(q26;p13). Cells harboring the t(3;12) translocation were found to exhibit a higher proliferative capacity than cells with pure trisomy 8, which is consistent with the role of the ETV6/MDS1/EVI1 fusion transcript in the development and progression of malignancy.

## Introduction

Chromosomal abnormalities are extremely common in malignancy, including leukemia. Acute myelogeneous leukemia (AML) has a wide variety of chromosomal rearrangements that involve specific regions (1–3). The 3q26 region encodes two proteins involved in AML, ectopic viral integration site 1 (EVI1) and myelodysplastic syndrome 1 (MDS1). Expression of the EVI1 gene has been found to correlate with accelerated cell growth of murine embryonic stem cells. By contrast, the combined effect of these two genes has been observed to play a transcriptional transactivator role, resulting in reduced cell growth (4).

The ETV6 gene, located at 12p13, encodes a transcription factor containing the 5′ helix-loop-helix dimerization motif and the 3′ ETS DNA-binding domain (5). To date, >40 partner cytobands have been identified to be associated with translocations involving ETV6 and ≥28 partner genes encoding protein tyrosine kinases, transcription factors or other proteins (5). The t(3;12)(q26.2;p13) translocation is a recurrent translocation involving ETV6. This translocation is relatively rare but specifically observed in myeloid malignancies, including myelodysplastic syndrome (MDS), AML and chronic myelogenous leukemia (CML) (6). It may also be associated with dysplasia of megakaryocytes, multilineage involvement and disease progression (7). Finally, the 3q26 locus is rearranged in inversion 3(q21;q26) syndrome, which represents an AML subtype showing dysmegakaryopoiesis, thrombocytosis and micromegakaryocytes (8).

At present, mutual translocation of MDS1/EVI1 and ETV6 has been observed in only two AML-M4 cases; secondary to myelodysplastic syndrome (MDS) and in CML in blast crisis (9,10). In the current case report, we describe the molecular and cytogenetic characterization of a *de novo* AML-M4 case with t(3;12)(q26;p13) and trisomy 8. Written informed consent was obtained from the patient.

## Case report

### Patient characteristics

A 63-year old female was diagnosed with AML-M4 in October 2011 due to loss of weight and fever. Hematological parameters were as follows: White blood cell count, 5.43×10^9^ cells/l; composed of 32.4% neutrophils, 24.4% lymphocytes, 38.5% monocytes, 0.61% eosinophils and 4.1% basophils. The platelet count was 1.32×10^9^ cells/l and hemoglobin levels were 9.61 g/dl. Serum LDH levels were 1,121 U/l (normal, ≤480 U/l). No treatment had been administered prior to the test. In December 2011, the patient succumbed to unknown causes whilst under treatment with 100 mg Cytosar.

### Methods

#### Chromosome analysis

Chromosome analysis using GTG-banding was performed according to standard procedures prior to chemotherapeutic treatment (11). A total of 20 metaphase cells derived from unstimulated bone marrow culture were analyzed. Karyotypes were described according to the International System for Human Cytogenetic Nomenclature (12).

#### Molecular cytogenetics

Fluorescence *in situ* hybridization (FISH), using the LSI BCR/ABL dual color dual fusion translocation and chromosome enumeration probes (CEPs) for chromosomes 3 and 12 (both Abbott Laboratories, Des Plaines, IL, USA), were applied according to the manufacturer’s instructions, together with the TEL/AML1 translocation dual fusion probe (Qbiogene, MP Biomedicales, Santa Ana, CA, USA) (11). A total of 20 metaphase spreads were analyzed, each using a fluorescence microscope (Axio Imager Z1; Zeiss, Oberkochen, Germany) equipped with appropriate filter sets to discriminate between a maximum of five fluorochromes and the counterstain DAPI. Image capturing and processing were performed using an ISIS imaging system (MetaSystems, Altlußheim, Germany).

#### Reverse transcription-polymerase chain reaction (RT-PCR)

RT-PCR was performed to investigate the expression of human ETV6/MDS1/EVI1 fusion transcripts. Total RNA was extracted from the diagnostic peripheral blood sample using the InviTrap RNA kit (Invitek, Berlin, Germany) according to the manufacturer’s instructions. cDNA was prepared from 5 μg total RNA with the Genequality BCR-ABL kit (AB Analitica, Padova, Italy) according to the manufacturer’s instructions. The primers used for ETV6/MDS1/EVI1 were as previously reported (13).

#### Flow cytometric immunophenotype

Immunophenotyping of leukemic blasts was performed as described previously (14). Briefly, flow cytometric analysis was performed using a general panel of fluorescent antibodies against the following antigens typical for different cell lineages and cell types: CD1a, CD2, CD3, CD4, CD5, CD8, CD10, CD11b, CD11c, CD13, CD14, CD15, CD16, CD19, CD20, CD22, CD23, CD32, CD33, CD34, CD38, CD41a, CD45, CD56, CD57, CD64, CD103, CD117, CD123, CD138, CD209, CD235a and CD243, as well as antibodies to κ and λ light Chains, IgD, sIgM and HLA-DR. All antibodies were purchased from BD Biosciences (San Jose, CA, USA). Samples were analyzed on a BD FACSCalibur™ flow cytometer. Autofluorescence, viability and isotype controls were included. Flow cytometric data acquisition and analysis were conducted by BD Cellquest™ Pro software.

### Results

**Figure 1:**
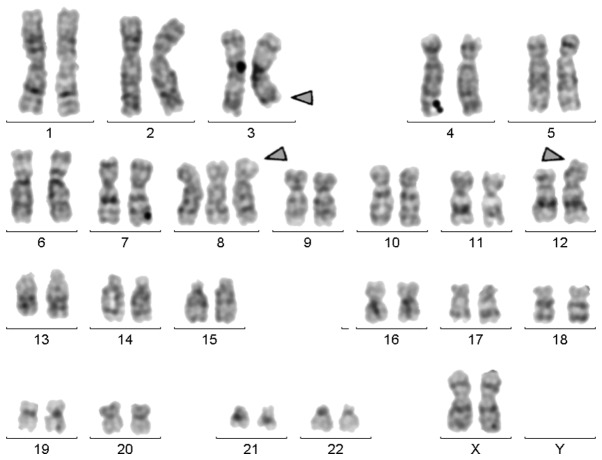
GTG-banding revealed the following karyotype: 47,XX,+8,t(3;12). All derivative chromosomes are highlighted by arrowheads.

**Figure 2:**
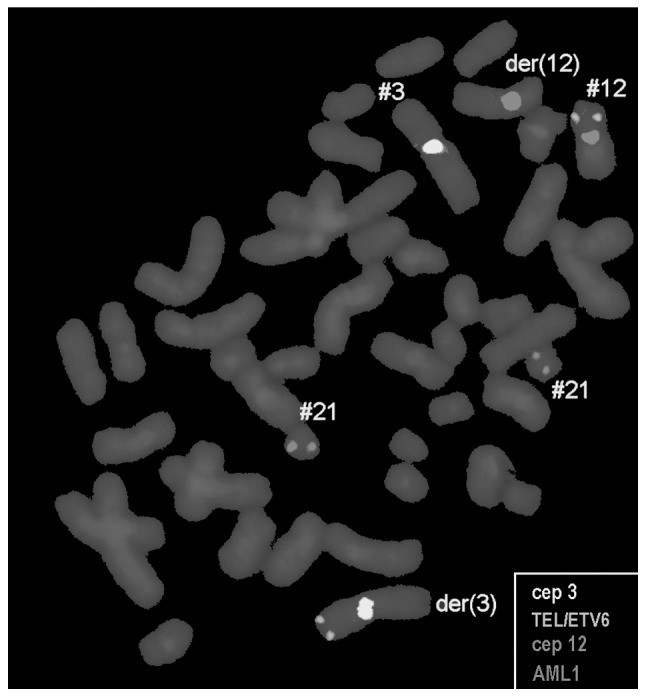
Metaphase fluorescence *in situ* hybridization using CEP(3) (#3) and CEP(12) (#12) probes, along with the TEL/AML1 translocation dual fusion probe, confirmed an involvement of chromosome 3 with chromosome 12 in this case. A TEL (ETV6) signal was located on der(3). CEP, chromosome enumeration probe; #, chromosome; der, derivative chromosome.

Karyotyping was performed prior to chemotherapy and the result was 47,XX,+8,t(3;12)[18]/47,XX,+8[2] (Fig. 1). This observation was further confirmed by molecular cytogenetic studies (Fig. 2). Dual-color-FISH using a CEP probe specific for chromosomes 3 and 12 was mixed with the TEL/AML1 translocation probe (Fig. 2). Thus, the following final karyotype was obtained: 47,XX,+8,t(3;12)(q26;p13)[18]/47,XX,+8[2].

**Figure 3:**
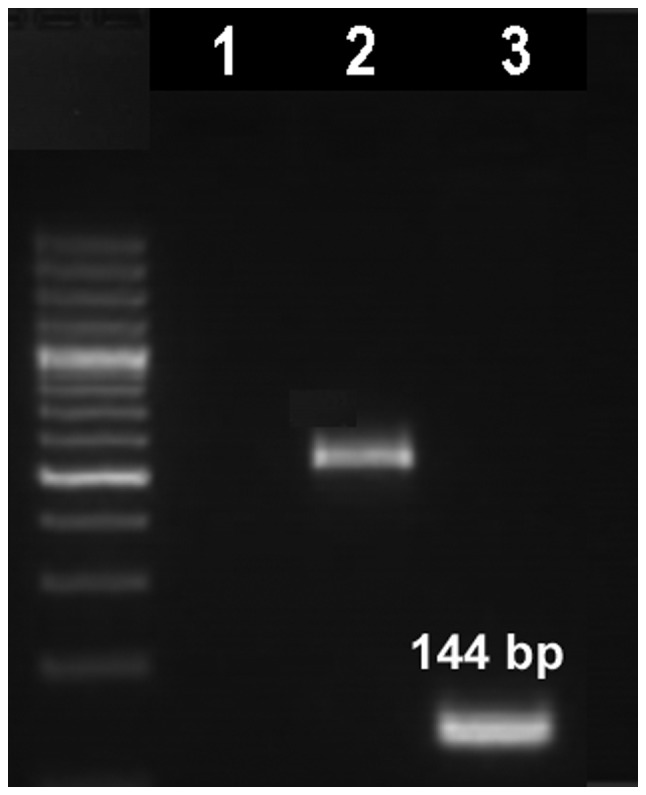
Gel electrophoresis of multiplex reverse transcription-polymerase chain reaction products from the present case. Lanes 1, negative control; 2, internal control (β2-microglobulin; 535 bp) and 3, ETV6-MDS1-EVI1 fusion transcript (144 bp). MDS1, myelodysplastic syndrome; EVI1, ectopic viral integration site 1.

The t(3;12) translocation was further studied by RT-PCR and the analysis revealed a typical fusion transcript of 144 bp in length, which confirmed the presence of an ETV6/MDS1/EVI1 fusion transcript (Fig. 3).

Immunophenotyping analysis of the peripheral blood showed that the abnormal cell population was CD45 (96%), CD33 (78%), CD13 (78%), CD18 (91%) and CD11c (55%), and that CD34 (32%), HLA-DR (58%), CD117 (32%), CD32 (26%), CD16 (54%), CD15 (20%) and CD235a (29%) were expressed heterogeneously. This immunophenotype is consistent with AML-M4 according to FAB classification.

## Discussion

The t(3;12)(q26;p13) translocation is a rare cytogenetic abnormality and has been previously reported in 45 cases of myeloid malignancies, including MDS, AML and CML (6). Two cases were described as AML-M4; one of them was initially a CML case with an additional trisomy 8 (6). To the best of our knowledge, the present case report is the first to observe a case of a AML-M4 with initial trisomy 8 and secondary developed t(3;12)(q26;p13).

The common chromosomal abnormalities in AML-M4 include monosomy 5 or del(5q), monosomy 7 or del(7q), trisomy 8, t(6;9)(p23;q34) and rearrangements involving the MLL gene mapped to 11q23 [del(11)(q23); t(9;11)(p22;q23), t(11;19)(q23;p13)] and core binding factor B (CBFβ) mapped to 16q22 [del(16)(q22), inv(16)(p13q22), t(16;16)(p13;q22)] (15). However, in the present case, trisomy 8 was observed. Trisomy 8 is the most frequent numerical aberration in AML, occurring at a frequency of 10–15%. A previous study reported that AML patients with trisomy 8 have poor outcomes and are not responsive to cytarabine-based therapy (15). In addition, a study reported that trisomy 8 confers an independent prognostic risk in AML (16).

The prognosis of AML with the t(3;12)(q26;p13) translocation has been reported to be poor, with a survival of only a few months. This is associated with treatment refractoriness and may reflect the fact that this AML is secondary to MDS with the presence of chromosome 7 abnormalities (4). This has a poor prognosis in general and is observed in the majority of t(3;12)(q26;p13) cases with chromosome 7 rearrangements. In three cases, resistance to therapy included treatment with allogeneic bone marrow transplantation (6,7,17). Notably, all three patients had an early relapse within a few months from transplantation (4).

EVI1 is a transcription factor with two zinc finger motifs and its acquisition is known to be a poor prognostic factor for AML (18). It is located on 3q26 and its aberrant expression is mainly mediated by the t(3;3)(q21;q26), inv(3)(q21q26), t(3;12)(q26;p13) and t(3;21)(q26;q22) genomic aberrations (19). These abnormalities lead to 3q21q26 syndrome, which is associated with thrombocytosis, megakaryocytic dysplasia, resistance to chemotherapy and poor prognosis (4–20).

The MDS1/EVI1 fusion generates a protein domain with homology to the positive regulatory domain of PRDI-BF1/Blimp-l, a transcriptional repressor of the interferon β gene and an inducer of genes that play a role in B-lymphocyte maturation (21).

In conclusion, the current case report presents a novel cytogenetic case of AML-M4 with initial trisomy 8 and a secondary t(3;12)(q26;p13), the latter having more proliferative capacity than cells with pure trisomy 8. The patient succumbed to unknown causes whilst under treatment. Therefore, we conclude that trisomy 8 with t(3;12)(q26;p13) has a negative prognosis.
